# Blockade of Autophagy Prevents the Progression of Hyperuricemic Nephropathy Through Inhibiting NLRP3 Inflammasome-Mediated Pyroptosis

**DOI:** 10.3389/fimmu.2022.858494

**Published:** 2022-03-02

**Authors:** Yan Hu, Yingfeng Shi, Hui Chen, Min Tao, Xun Zhou, Jinqing Li, Xiaoyan Ma, Yi Wang, Na Liu

**Affiliations:** Department of Nephrology, Shanghai East Hospital, Tongji University School of Medicine, Shanghai, China

**Keywords:** autophagy, pyroptosis, hyperuricemic nephropathy, NLRP3 inflammasome, cathepsin B

## Abstract

Hyperuricemia has become a common metabolic disease, and is a risk factor for multiple diseases, including chronic kidney disease. Our recent study indicated that following persistent uric acid stimulation, autophagy was activated in rats of hyperuricemic nephropathy (HN) and facilitated the development of renal fibrosis. Nevertheless, the potential mechanism by which autophagy promoted the progression of HN is still not fully elucidated. Thus, in the current study, we investigated the mechanisms of autophagy inhibition on the development of HN. Our data showed that autophagy was activated in human renal tubular cell lines (HK-2) exposure to uric acid. Inhibition of autophagy with 3-methyladenine (3-MA) and transfected with Beclin-1 siRNA prevented uric acid-induced upregulation of α-SMA, Collagen I and Collagen III in HK-2 cells. Moreover, uric acid upregulated autophagy *via* promoting the p53 pathway. *In vivo*, we showed that hyperuricemic injury induced the activation of NLRP3 inflammasome and pyroptosis, as evidenced by cleavage of caspase-1 and caspase-11, activation of gasdermin D (GSDMD) and the release of IL-1β and IL-18. Treatment with autophagy inhibitor 3-MA alleviated aforementioned phenomenon. Stimulation with uric acid in HK-2 cells also resulted in NLRP3 inflammasome activation and pyroptotic cell death, however treatment with 3-MA prevented all these responses. Mechanistically, we showed that the elevation of autophagy and degradation of autophagolysosomes resulted in the release of cathepsin B (CTSB), which is related to the activation of NLRP3 inflammasome. CTSB siRNA can inhibit the activation of NLRP3 inflammasome and pyroptosis. Collectively, our results indicate that autophagy inhibition protects against HN through inhibiting NLRP3 inflammasome-mediated pyroptosis. What’s more, blockade the release of CTSB plays a crucial role in this process. Thus, inhibition of autophagy may be a promising therapeutic strategy for hyperuricemic nephropathy.

## Introduction

With the rapid development of the world economic and the improvement of lifestyle, the prevalence of hyperuricemia is remarkably increasing all over the world (1, 2). About 75% of uric acid is excreted by the kidneys, and chronic uric acid stimulation to the kidney results in renal tubule-interstitial fibrosis, eventually causes hyperuricemic nephropathy (HN) (3). The features of HN includes crystal kidney stones, chronic interstitial nephritis, and renal fibrosis (4), which become an important public health issue (5). To date, the treatment of HN mainly focuses on the drugs that suppress the production of uric acid and promote the excretion of uric acid, such as the first-line drugs allopurinol and benzbromarone respectively. However, their clinical applications are limited by the severe side effects, such as hepatotoxicity, nephrotoxicity, and Stevens-Jonson syndrome (6), using uric acid-lowering agents in HN is still controversial (7). Hence, it is imperative to search appropriate therapeutic strategy for HN.

Recently, we found that following chronic hyperuricemic damage, autophagy was activated in HN rats and closely associated with renal fibrosis (8). Autophagy is a process that identifies damaged organelles or misfolded proteins and degrades them by fusion with lysosomal compartments (9). During autophagy, cells formed double-membraned vesicles and autophagosomes, that sequester proteins or organelles for delivery to lysosome (10). Autophagy plays an important role in maintaining the homeostasis, and it can regulate the development and differentiation of specific cells, such as adipocytes and lymphocytes (11). However, studies have reported that autophagy is also involved in pathological mechanisms (12, 13). Using autophagy inhibitor 3-methyladenine (3-MA), our previous research has indicated that inhibition of autophagy alleviated HN in an adenine (0.1 g/kg) and potassium oxonate (1.5 g/kg)-induced rat model and an *in vitro* model of uric acid stimulated cultured rat renal interstitial fibroblasts (NRK-49F). We demonstrated that inhibition of autophagy with 3-MA suppresses activation of renal interstitial fibroblasts and production of extracellular matrix components in NRK-49F and in HN rats. Moreover, we also found that autophagy promoted the progression of HN by activation of the pro-fibrosis cytokines/growth receptors, augment the responses of inflammation and mediate the G2/M arrest (8). Nevertheless, the potential mechanism of autophagy promoted the deterioration of HN is unclear so far, further studies are needed to elucidate the mechanisms by which blockade of autophagy ameliorates the progression of HN.

Currently, more and more studies have shown that autophagy plays an important role in inflammatory responses. Since pyroptosis is a form of inflammatory cell death, the relationship between autophagy and pyroptosis has aroused interests. Pyroptosis is a cell death mode characterized by plasma membrane rupture, cytoplasmic swelling, osmotic lysis, DNA cleavage, and the release of a large number of pro-inflammatory cytokines (14). There are two pathways to induce pyroptosis: the classical caspase-1 dependent pathway and the nonclassical caspase-11 dependent secretory pathway (15, 16). Accumulating evidence demonstrates that inflammasome activation plays a critical role for pyroptosis. The nucleotide binding and oligomerization domain-like receptor family pyrin domain-containing 3 (NLRP3) inflammasome, is the most widely investigated among all NLR-related inflammasomes (17). The activation of NLRP3 will recruits and cleaves pro-caspase-1 into its active forms. Once being active, the caspase-1 has a specific structure of heterotetramers that regulate proteolytic processes of inflammatory and inflammatory cytokines, such as IL-1β and IL-18 (18). The pyroptosis-driven key role in gasdermin family, gasdermin D (GSDMD) in particular, is the most widely investigated. The activated form of both caspase-1 and caspase-11 can cleave GSDMD and separate the N-terminal fragment from the C-terminal fragment (19). The cell membrane forms membrane pores thereby inducing more inflammatory cytokines release (20). At present, the unequivocal upstream activation mechanism of NLRP3 inflammasome has not yet been clarified. Nevertheless, a study showed that the NLRP3 inflammasome was regulated by cathepsin B (CTSB) (21). In addition, the release of CTSB is induced by autophagy. In briefly, the upregulation of autophagy and degradation of autophagolysosomes will lead to the release of CTSB, which also related to the stimulation of NLRP3 inflammasome (22, 23). However, whether uric acid induced autophagy, release of CTSB, activation of NLRP3 inflammasome and pyroptosis lead to HN have not been investigated.

In the current study, we revealed the activation of autophagy in uric acid-stimulated human renal tubular cell lines (HK-2), and explored the mechanisms by which inhibition of autophagy ameliorates the development of HN induced by feeding a mixture of adenine and potassium oxonate.

## Materials and Methods

### Antibodies and Reagents

3-MA was purchased from Selleckchem (Houston, TX, USA). Antibodies to Beclin-1 (#3738), Atg7 (#2631), and p53 (#2527) were purchased from Cell Signaling Technology (Danvers, MA, USA). Antibodies to GAPDH (sc-32233), Collagen I (sc-28654), and Caspase-11 (sc-374615) were purchased from Santa Cruz Biotechnology (San Diego, CA, USA). Antibodies to NLRP3 (ab214185), and Caspase-1 (ab179515) were purchased from Abcam (Cambridge, MA, USA). Antibodies to Collagen III (GB11023), and IL-1β (GB11113) were purchased from Servicebio (Wuhan, China). Antibodies to IL-18 (A16737) and GSDMD (A20197) were purchased from ABclonal Biotech (Shanghai, China). Antibody to cathepsin B (12216-1-AP) was purchased from Proteintech Group (Chicago, IL, USA). Anti-mouse secondary antibody (A0216), and anti-rabbit secondary antibody (A0208) were purchased from Beyotime Institute of Biotechnology (Shanghai, China). Beclin-1 siRNA and GP-transfect-Mate (G04009) were purchased from GenePharma (Shanghai, China). Antibody to α-SMA (A2547), DMSO and all other chemicals were obtained from Sigma-Aldrich (St. Louis, MO, USA).

### HN Rat Model

The HN model was established in male Sprague-Dawley rats (6-8 weeks old, Shanghai Super-B&K Laboratory Animal Corp. Ltd, Shanghai, China) that weighed 200-220g. The animals were housed at the Experimental Animal Center of Tongji University under a 12 h light-dark cycle with food and water supplied ad libitum. The HN model was established as described in our previous study (8). Rats were injected intraperitoneally with 3-MA 15mg/kg in warmed saline daily in order to explore the effect of 3-MA on HN. Rats were randomly divided into four groups, each group including six rats: (1) The rats in control group were given an equivalent amount of saline by gavage and injected with an equivalent amount of saline intraperitoneally; (2) The rats in sham+3-MA group were given an equivalent amount of saline by gavage and injected with an equivalent amount of 3-MA intraperitoneally; (3) The rats in HN group were given a mixture of adenine (0.1 g/kg) and potassium oxonate (1.5 g/kg) by gavage and injected with an equivalent amount of saline intraperitoneally,; (4) The rats in HN+3-MA group were given a mixture of adenine (0.1 g/kg) and potassium oxonate (1.5 g/kg) by gavage and injected with an equivalent amount of 3-MA intraperitoneally. At the end of the experimental period, all animals were killed by exsanguination under anesthesia with inhaled 5% isoflurane in room air and the kidney was collected for the following experiments. All the animal experiments were conducted with approval from the Institutional Animal Care and Use Committee at Tongji University.

### Cell Culture and Treatments

Human tubular epithelial cells (HK-2) were attained from ATCC (Manassas, VA). Cells were cultured in a 1:1 mixture of Dulbecco’s modified Eagle’s medium (DMEM) and F-12 containing 10% fetal bovine serum (FBS), 1% penicillin-streptomycin in an atmosphere of 5% CO_2_ and 95% air at 37°C. Before starting the formal experiments, we passed the primary cells for three generations in order to obtain a stable phenotype. To investigate the effect of 3-MA in uric acid-induced tubular cell injury, subconfluent HK-2 cells were starved for 24 hours in DMEM medium containing 0.5% FBS and then exposed to uric acid (UA, 800 μM) in the presence of 3-MA (0, 1, 5 and 10 mM) for 36 hours. After stimulation for 36 hours, cells were harvested for further analysis. All of the *in vitro* experiments were repeated no less than three times.

### siRNA Transfection

The small interfering (si) RNA oligonucleotides targeted specially for p53, Beclin-1, CTSB, and negative control (NC) siRNA which chemically synthesized by GenePharma (Shanghai, China) were used in this study. The sequence of p53 siRNA is 5′-GACUCCAGUGGUAAUCUACTT-3′ (sense strand) and 5′-GUAGAUUACCACUGGAGUCTT-3′ (antisense strand). The sequence of Beclin-1 siRNA is 5′-CAGUUUGGCACAAUCAAUATT-3′ (sense strand) and 5′-UAUUGAUUGUGCCAAACUGTT-3′ (antisense strand). The sequence of CTSB siRNA is 5′-ACAAGCACUACGGAUACAUTT-3′ (sense strand) and 5′-AUGUAUCCGUAGUGCUUGUTT-3′ (antisense strand). The sequence of NC siRNA is 5′-UUCUCCGAACGUGUCACGUTT-3′ (sense strand) and 5′-ACGUGACACGUUCGGAGAATT-3′ (antisense strand). Briefly, HK-2 cells were grown in 6-well plates and transiently transfected with siRNA at 60-80% confluence at a final concentration of 5 nM using GP-transfect-Mate (GenePharma, Shanghai, China) according to the manufacturer’s instructions. After 6 hours transfection, the cells were incubated with fresh medium alone or administrated with uric acid (800 μM) for an additional 36 hours before being harvested for the further experiments.

### Immunoblot Analysis

Cells were washed three times with ice-cold PBS and harvested in RIPA buffer mixed with PMSF and phosphatase inhibitor on ice. The supernatants were collected after centrifugation at 12,000 g for 15 min at 4°C. In addition, the kidney tissue samples were homogenized with cell lysis buffer and with PMSF and phosphatase inhibitor. Proteins were separated by SDS-PAGE and transferred to 0.2 μm nitrocellulose membranes. After incubation with 5% nonfat milk for 1 hour at room temperature, the membranes were incubated with primary antibodies overnight at 4°C and then incubated with appropriate horseradish peroxidase-conjugated secondary antibodies for 1 hour at room temperature. Bound antibodies were visualized by chemiluminescence detection. Densitometry analysis of immunoblot results was conducted by using Image J software (National Institutes of Health, Bethesda, MD).

### Immunohistochemical Staining

Formalin-fixed kidneys were imbedded in paraffin and prepared in 3-μm-thick sections. Sections were de-paraffinized and rehydrated, immersed in citrate buffer and heated in a microwave for retrieval of antigens and quenched with 3% H_2_O_2_. Sections were incubated with primary antibodies overnight at 4°C and then incubated with appropriate horseradish peroxidase-conjugated secondary antibodies for 1 hour at room temperature. For quantifications, 10 random visual fields were analyzed per kidney section. The positive area was calculated with Image J software (National Institutes of Health, Bethesda, MD).

### Immunofluorescence Staining

Formalin-Fixed Paraffin-Embedded sections (3 μm) were rehydrated and incubated with primary antibodies against p53, GSDMD, IL-1β, and IL-18 and then Texas Red- or FITC-labeled secondary antibodies (Invitrogen).

After treatments, HK-2 cells were plated on coverslips and then fixed in 4% paraformaldehyde for 10 minutes. Cells were permeabilized by 0.25% Triton X-100 in PBS for 10 minutes. After blocking with 10% normal goat serum, cells were incubated with primary antibodies against NLRP3, Caspase-11, GSDMD, IL-1β, IL-18, or CTSB followed by secondary antibodies. Finally, cells were stained with DAPI and mounted. Images were acquired using Fluorescence Microscope (Leica, DM3000).

### Transmission Electron Microscope

After HK-2 cells were treated in accordance with the aforementioned cell culture and treatments and reached confluence, cells were collected from each group for standard transmission electron microscope (TEM) processing to observe the morphology of autophagosome. Multiple autophagic structures such as phagophore, autophagosome, and autolysosome in HK-2 cells were observed at high magnification from each cell and digital images with scale bars were taken.

### Statistical Analysis

Data depicted in graphs expressed as means ± SEM for each group. The comparisons between two groups were analyzed by Student’s t-test and one-way analysis of variance was used for comparisons of multiple groups. Statistically significant differences between mean values were marked in each graph. *P*<0.05 was considered statistically significant.

## Results

### Exposure of HK-2 Cells to Uric Acid Results in the Activation of Autophagy and the Upregulation of α-SMA, Collagen I and Collagen III

**Figure 1:**
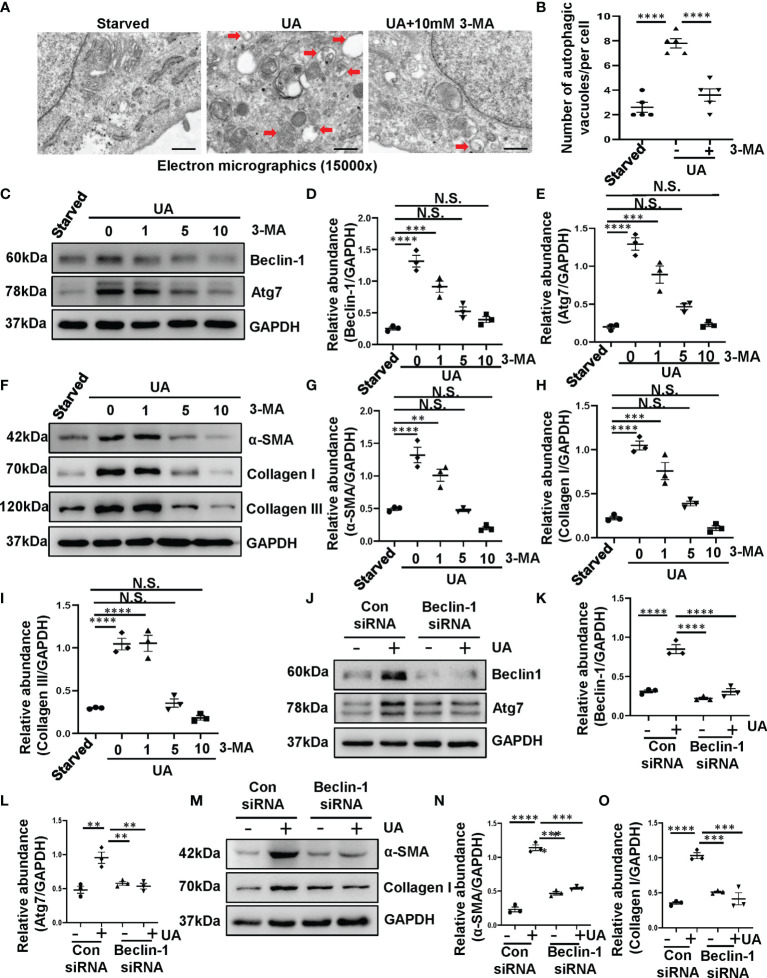
Exposure of HK-2 cells to uric acid results in the activation of autophagy and the upregulation of α-SMA, Collagen I and Collagen III. **(A)** Transmission electron microscopy showed the ultrastructural feature of autophagosome (Red arrows) in HK-2 cells following uric acid (800 μM) stimulation in the presence/absence of 3-MA. **(B)** Quantitation analysis of the number of autophagic vacuoles per cell was performed. **(C)** Western blot was conducted to evaluate the protein level of Beclin-1, Atg7 and GAPDH in HK-2 cell lysates. **(D, E)** Scatter plots showing the densitometry analysis of Beclin-1 and Atg7 normalized by GAPDH. **(F)** Western blot was conducted to evaluate the protein level of α-SMA, Collagen I, Collagen III and GAPDH in HK-2 cell lysates. **(G–I)** Scatter plots showing the densitometry analysis of α-SMA, Collagen I, Collagen III normalized by GAPDH. **(J)** Western blot was conducted to evaluate the protein level of Beclin-1, Atg7 and GAPDH in HK-2 cell lysates. **(K, L)** Scatter plots showing the densitometry analysis of Beclin-1 and Atg7 normalized by GAPDH. **(M)** Western blot was conducted to evaluate the protein level of α-SMA, Collagen I and GAPDH in HK-2 cell lysates. **(N, O)** Scatter plots showing the densitometry analysis of α-SMA and Collagen I normalized by GAPDH. Data are expressed as mean ± SEM. **P<0.01; ***P<0.001; ****P<0.0001. N.S., statistically not significant, with the comparisons labeled. Scale bars in **(A)** = 500 nm.

Our previous study has revealed that the increased expression of LC3II/I and Beclin-1 were observed in the kidney of hyperuricemic injury rats, and numerous autophagic vacuoles appeared in proximal tubular cells *in vivo* (8). It suggested that hyperuricemia can induce the activation of autophagy. In addition, kidneys with HN displayed severely structural damage as characterized by glomerulosclerosis, tubular dilation, epithelial atrophy, interstitial expansion, and collagen accumulation. Inhibition of autophagy with 3-MA decreased the deposition of extracellular matrix components. However, whether uric acid induced the activation of autophagy *in vitro* is still unknown, and how autophagy effects on renal tubular cells have not been elucidated. Therefore, we performed *in vitro* experiments on HK-2 cells with the stimulation of uric acid. As shown in **Figure 1A**, images from TEM indicated multiple of autophagy-related vacuoles such as autophagosome and autolysosome were observed in uric acid-stimulated HK-2 cells compared to control cells. Treatment with 3-MA remarkably inhibited the autophagic activity (**Figures 1A, B**). Uric acid also induced a significant upregulation of Beclin-1 and Atg7, two autophagy related proteins, 3-MA dose-dependently suppressed these responses (**Figures 1C–E**). Reduction of Beclin-1 expression by its specific siRNA also decreased uric acid-stimulated expression of Atg7 (**Figures 1J–L**). Since epithelial-to-mesenchymal transition (EMT) is involved in the progression of renal fibrosis (24), we found that compared with serum-starved HK-2 cells, uric acid-exposed HK-2 cells expressed higher protein levels of α-SMA, Collagen I, and Collagen III, three mesenchymal markers. Inhibition of autophagy with 3-MA significantly reduced the expression of α-SMA, Collagen I, and Collagen III (**Figures 1F–I**). Consistent with this result, knocking down of Beclin-1 also blocked the expression of α-SMA and Collagen I (**Figures 1M–O**). Taken together, these results indicated that autophagy was activated in the uric acid-stimulated HK-2 cells, which is essential for the occurred of EMT in tubular epithelial cells.

### Uric Acid Induces Autophagy Mediated by p53 Signaling Pathway

**Figure 2:**
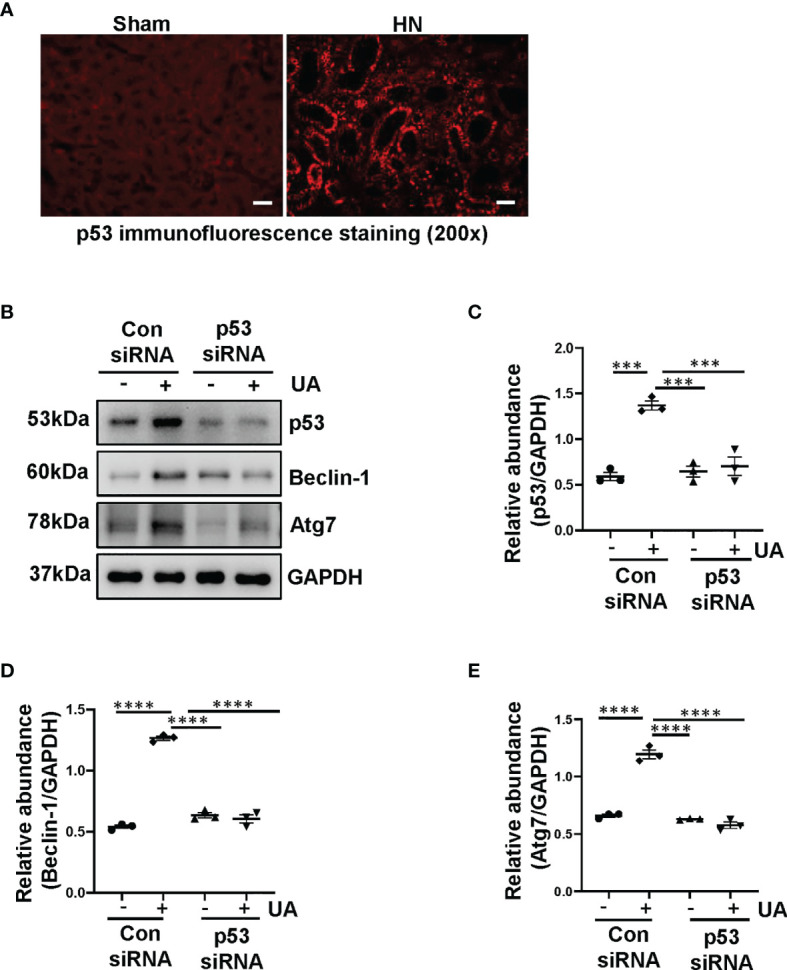
Uric acid induces autophagy mediated by p53 signaling pathway. **(A)** Photomicrographs illustrating immunofluorescence staining of p53. **(B)** Western blot was conducted to evaluate the protein level of p53, Beclin-1, Atg7 and GAPDH in HK-2 cell lysates. **(C–E)** Scatter plots showing the densitometry analysis of p53, Beclin-1 and Atg7 normalized by GAPDH. Data are expressed as mean ± SEM. ***P<0.001; ****P<0.0001. Scale bars in **(A)** = 50 μm.

Based on the aforementioned role of uric acid in the activation of autophagy, we further investigated the mechanism involved. It is illustrated that p53 triggered the activation of autophagy in response to DNA damage (25), we thus examined whether uric acid upregulated autophagy through p53 signaling pathway. We established a rat model of HN induced by adenine and potassium oxonate for 3 weeks. As shown in **Figure 2A**, compared to the sham rats, HN rats displayed increased expression of p53 in the kidney. To further verify the role of p53 on the activation of autophagy, we examined the effect of p53 knockdown in HK-2 cells exposure to uric acid by using siRNA specifically targeting p53. As demonstrated in **Figures 2B–E**, reduction of p53 expression by its specific siRNA decreased uric acid stimulated expression of Beclin-1 and Atg7. These results demonstrated that uric acid triggered autophagy may be mediated by p53 signaling pathway.

### Inhibition of Autophagy Prevents the Activation of NLRP3 Inflammasome Both in HN Rats and in HK-2 Cells

**Figure 3:**
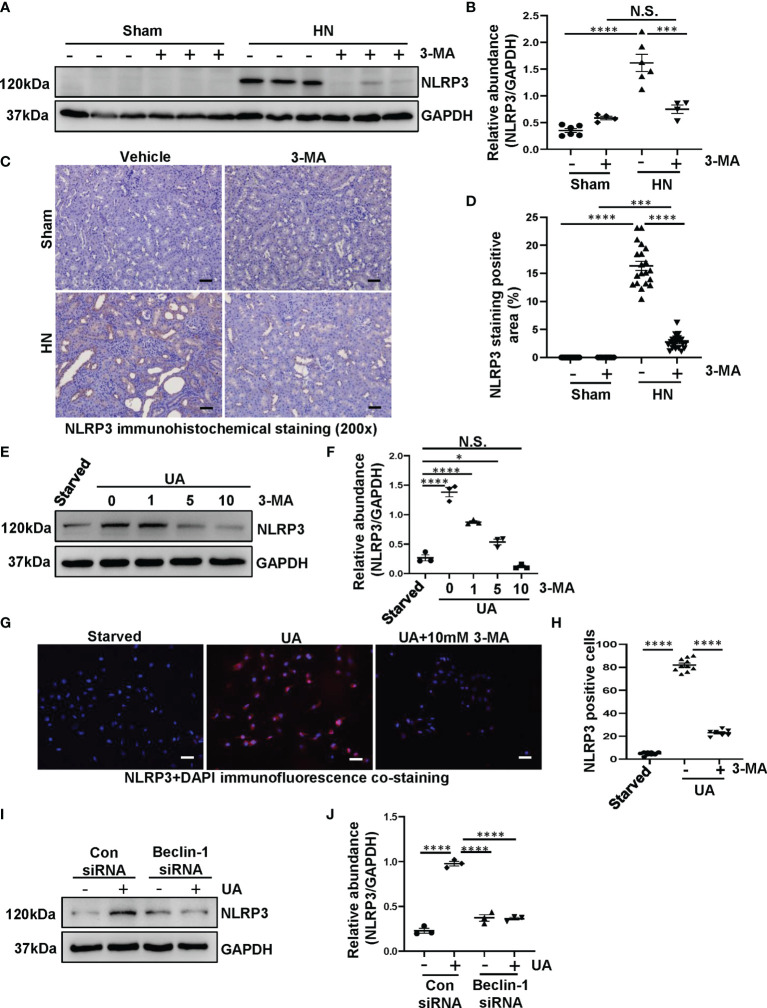
Inhibition of autophagy prevents the activation of NLRP3 inflammasome both in HN rats and in HK-2 cells. **(A)** Western blot was conducted to evaluate the protein level of NLRP3 and GAPDH in the kidney tissue lysates. **(B)** Scatter plot showing the densitometry analysis of NLRP3 normalized by GAPDH. **(C)** Immunohistochemistry staining was used to detect the level of NLRP3. **(D)** Positive area of NLRP3. **(E)** Western blot was conducted to evaluate the protein level of NLRP3 and GAPDH in HK-2 cell lysates. **(F)** Scatter plot showing the densitometry analysis of NLRP3 normalized by GAPDH. **(G)** Immunofluorescence co-staining was used to detect the level of NLRP3. **(H)** The count of NLRP3-positive cells. **(I)** Western blot was conducted to evaluate the protein level of NLRP3 and GAPDH in HK-2 cell lysates. **(J)** Scatter plot showing the densitometry analysis of NLRP3 normalized by GAPDH. Data are expressed as mean ± SEM. *P<0.05; ***P<0.001; ****P<0.0001. N.S., statistically not significant, with the comparisons labeled. All scale bars = 50 μm.

Hyperuricemia can trigger inflammatory responses in the kidney, and the inflammasome family has several members and behaves a magnificent effect on inflammatory responses. Particularly, NLRP3 inflammasome is considered to be a pivotal component of inflammation (26). Recently, emerging studies have demonstrated that there is an interaction between autophagy and NLRP3 inflammasome, more importantly, the interaction plays a crucial role in metabolic diseases (27). However, whether autophagy can activate the NLRP3 inflammasome in HN is still unknown. Thus, we get down to examine the effect of autophagy inhibition on the activation of NLRP3 inflammasome. As shown in **Figures 3A, B**, NLRP3 was significantly increased in HN rats, while administration with 3-MA resulted in the reduction of NLRP3. Immunohistochemistry staining also demonstrated that the expression level of NLRP3 was increased in the kidney of HN rats, which is contrary to that in sham rats. Treatment with 3-MA largely inhibited the expression as indicated by reduced staining positive areas (**Figures 3C, D**).

To further investigate the interplay between autophagy and NLRP3 inflammasome, we used both 3-MA and Beclin-1 siRNA to inhibit autophagy for *in vitro* studies. Exposure of HK-2 cells to uric acid resulted in increased expression of NLRP3, treatment with 3-MA inhibited the upregulation of NLRP3, which occurred in a dose-dependent manner (**Figures 3E, F**). The immunofluorescence staining was used to evaluate the expression and localization of NLRP3 in response to uric acid. We found that the cells were positively stained for NLRP3 in the cytoplasm stimulated by uric acid, treatment with 10 mM 3-MA significantly decreased the expression of NLRP3 (**Figures 3G, H**). In consistent with this result, immunoblotting analysis indicated that inhibition of autophagy with Beclin-1 siRNA was also impede the NLRP3 overexpression (**Figures 3I, J**). Taken together, these results suggest that inhibition of autophagy prevents the activation of NLRP3 inflammasome both *in vivo* and *in vitro*.

### Administration of 3-MA Inhibits the Process of Pyroptosis in a Rat Model of HN

**Figure 4:**
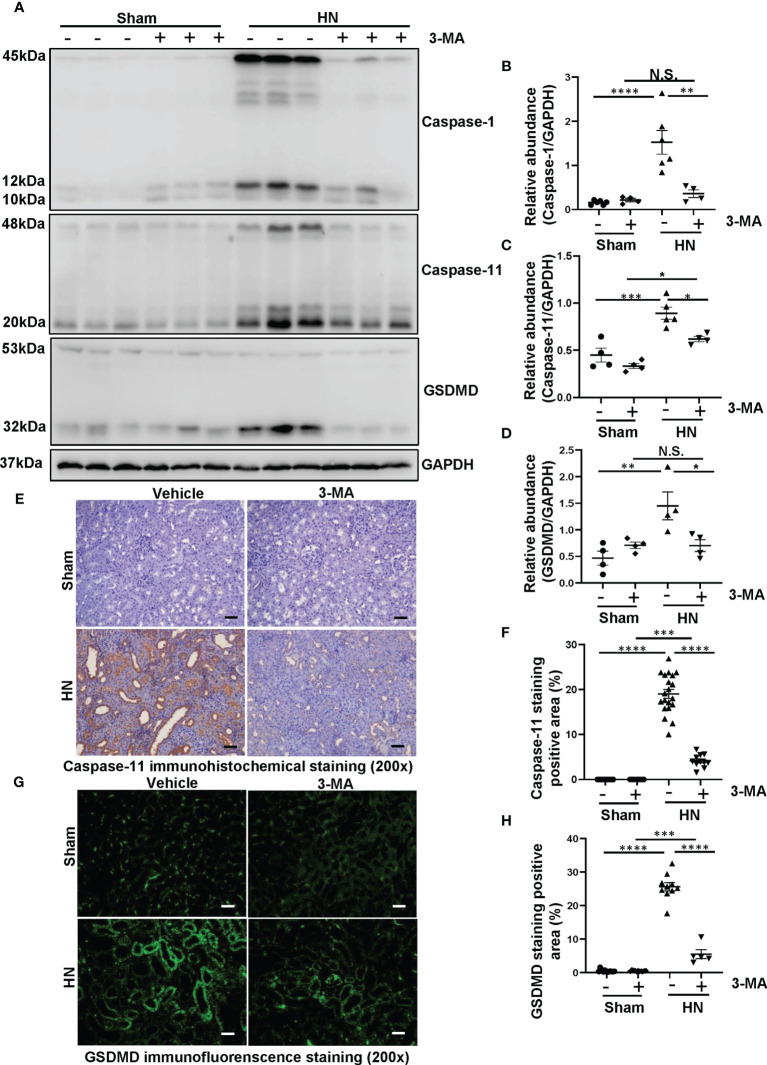
Administration of 3-MA inhibits the process of pyroptosis in a rat model of HN. **(A)** Western blot was conducted to evaluate the protein level of caspase-1, caspase-11, GSDMD and GAPDH in the kidney tissue lysates. **(B–D)** Scatter plots showing the densitometry analysis of caspase-1, caspase-11 and GSDMD normalized by GAPDH. **(E)** Immunohistochemistry staining was used to detect the level of caspase-11. **(F)** Positive area of caspase-11. **(G)** Immunofluorescence staining was used to detect the level of GSDMD. **(H)** Positive area of GSDMD. Data are expressed as mean ± SEM. *P<0.05; **P<0.01; ***P<0.001; ****P<0.0001. N.S., statistically not significant, with the comparisons labeled. All scale bars = 50 μm.

Shao et al. demonstrated that NLRP3 inflammasome leads to the activation of pyroptosis (28). In addition, a large number of studies indicate that a close relationship between autophagy and pyroptosis (29, 30). To investigate the role of autophagy in the NLRP3 inflammasome-mediated pyroptosis in HN, we adopted autophagy inhibitor 3-MA to verify the relationship among them. As shown in **Figures 4A–D**, proteins level of caspase-1, caspase-11, and GSDMD markedly increased in HN rats, pretreatment with 3-MA dramatically reduced the elevated expression of caspase-1, caspase-11, and GSDMD in the HN kidney. Immunohistochemistry of caspase-11 demonstrated that caspase-11 was predominantly localized in tubular epithelial cells in injured kidneys associated with HN, and 3-MA reduced the number of caspase-11-positive cells (**Figures 4E, F**). Moreover, immunofluorescence staining of GSDMD further revealed that GSDMD was primarily located in renal tubular epithelial cells and highly expressed in injured kidneys (**Figures 4G, H**). Thus, our results implied hyperuricemic induced autophagy and activated the NLRP3 inflammasome-mediated pyroptosis, and treatment with 3-MA was effective in ameliorating this responses.

### Inhibition of Autophagy Prevents the Process of Pyroptosis in HK-2 Cells Exposed to Uric Acid

**Figure 5:**
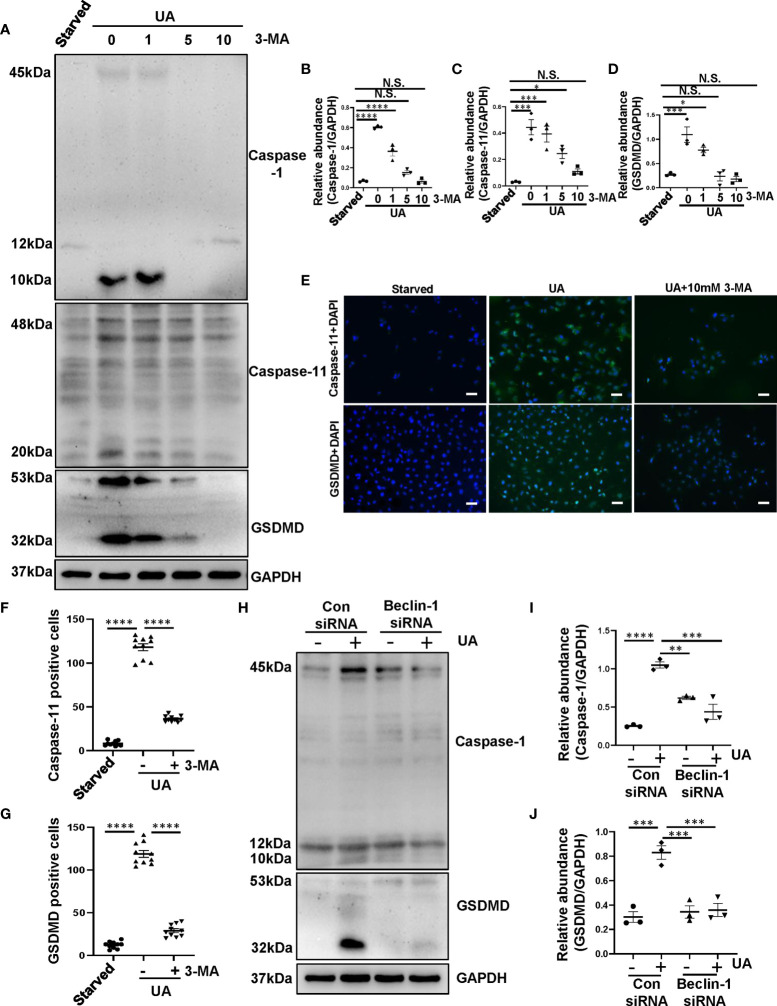
Inhibition of autophagy prevents the process of pyroptosis in HK-2 cells exposed to uric acid. **(A)** Western blot was conducted to evaluate the protein level of caspase-1, caspase-11, GSDMD and GAPDH in HK-2 cell lysates. **(B–D)** Scatter plots showing the densitometry analysis of caspase-1, caspase-11 and GSDMD normalized by GAPDH. **(E)** Photomicrographs illustrating immunofluorescence of caspase-11 and GSDMD, respectively, costained with DAPI. **(F, G)** Positive cells of caspase-11 and GSDMD were quantitatively analyzed. **(H)** Western blot was conducted to evaluate the protein level of caspase-1, GSDMD and GAPDH in HK-2 cell lysates. **(I, J)** Scatter plots showing the densitometry analysis of caspase-1 and GSDMD normalized by GAPDH. Data are expressed as mean ± SEM. *P<0.05; **P < 0.01.; ***P<0.001; ****P<0.0001. N.S., statistically not significant, with the comparisons labeled. All scale bars = 50 μm.

To further determine the role of autophagy in activating pyroptosis, we used autophagy inhibitor 3-MA as well as knockdown of Beclin-1 targeted by siRNA to inhibit autophagy for *in vitro* studies. Uric acid was found to induce a significant upregulation of caspase-1, caspase-11, and GSDMD, pretreatment with 3-MA dose-dependently reduced expression level of caspase-1, caspase-11 and GSDMD (**Figures 5A–D**). Immunofluorescence assay further confirmed that the expression of caspase-11 and GSDMD significantly increased in response to uric acid, treatment with 3-MA reduced the expression of caspase-11 and GSDMD in HK-2 cells (**Figures 5E–G**). As expected, knockdown of Beclin-1 reduced expression levels of caspase-1 and GSDMD (**Figures 5H–J**), which performed the same effects with inhibitor. Collectively, these data further conform that 3-MA inhibits the process of pyroptosis in HK-2 cells exposed to uric acid.

### Inhibition of Autophagy With 3-MA Prevents the Release of IL-1β and IL-18 Both in HN Rats and in HK-2 Cells

**Figure 6:**
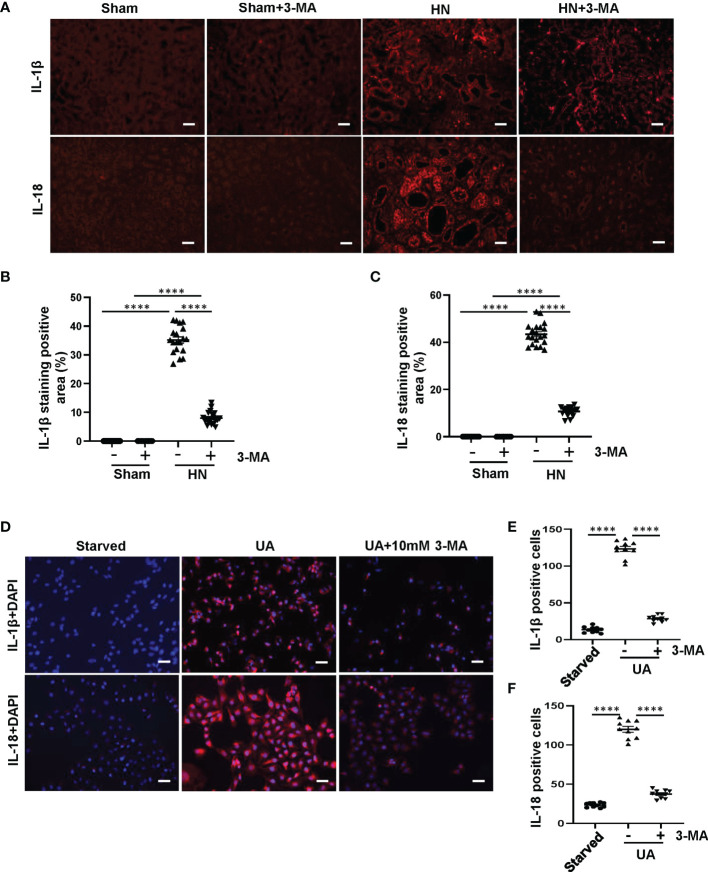
Inhibition of autophagy with 3-MA prevents the release of IL-1β and IL-18 both in HN rats and in HK-2 cells. **(A)** Photomicrographs illustrating immunofluorescence of IL-1β and IL-18 from kidney tissues. **(B, C)** Positive areas of IL-1β and IL-18 were quantitatively analyzed. **(D)** Photomicrographs illustrating immunofluorescence of IL-1β and IL-18, respectively, costained with DAPI. **(E, F)** Positive cells of IL-1β and IL-18 were quantitatively analyzed. ****P<0.0001. All scale bars = 50 μm.

After the process of pyroptosis, the freed-out N-terminal domain of GSDMD further regulates Pannexin and Potassium iron efflux, causes osmotic potential disruption, cell swelling and lysis, as well as releases of inflammatory cytokines such as IL-1β and IL-18 (31). To evaluate whether autophagy plays a crucial role in mediating the release of inflammatory cytokines after pyroptosis, we examined the effect of autophagy inhibition on the release of IL-1β and IL-18 *in vivo* and *in vitro*. Immunofluorescence staining showed that IL-1β and IL-18 were mainly located in the cytoplasm of renal tubules and highly expressed in the kidney of HN rats. 3-MA treatment inhibited the expression of IL-1β and IL-18 (**Figures 6A–C**). We further verified the role of autophagy in regulating the release of inflammatory cytokines in HK-2 cells exposed to uric acid. We found that after 36 hours uric acid stimulation, the cells were positively stained for IL-1β and IL-18 in the cytoplasm. Pretreatment with 10 mM 3-MA significantly reduced their expression (**Figures 6D–F**). All in all, these results indicate that inhibition of autophagy with 3-MA prevents the release of IL-1β and IL-18 both *in vivo* and *in vitro*.

### Uric Acid-Induced NLRP3 Inflammasome Activation and Pyroptosis Are Regulated by CTSB

**Figure 7:**
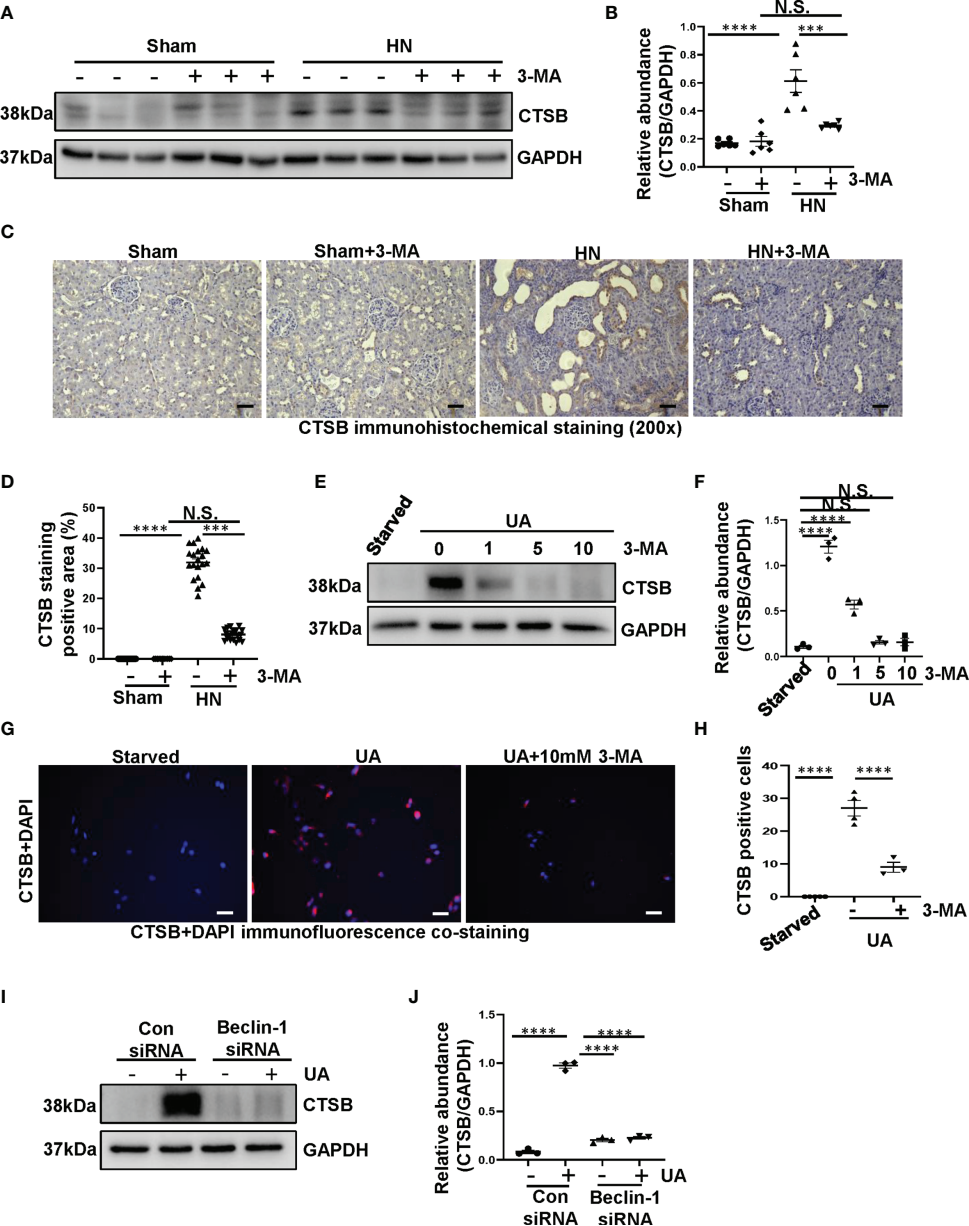
Uric acid upregulated the expression of CTSB both in HN rats and in HK-2 cells. **(A)** Western blot was conducted to evaluate the protein level of CTSB and GAPDH in the kidney tissue lysates. **(B)** Scatter plot showing the densitometry analysis of CTSB normalized by GAPDH. **(C)** Immunohistochemistry staining was used to detect the level of CTSB. **(D)** Positive area of CTSB. **(E)** Western blot was conducted to evaluate the protein level of CTSB and GAPDH in HK-2 cell lysates. **(F)** Scatter plot showing the densitometry analysis of CTSB normalized by GAPDH. **(G)** Immunofluorescence staining was used to detect the level of CTSB. **(H)** The count of CTSB-positive cells. **(I)** Western blot was conducted to evaluate the protein level of CTSB and GAPDH in HK-2 cell lysates. **(J)** Scatter plot showing the densitometry analysis of CTSB normalized by GAPDH. Data are expressed as mean ± SEM. ***P<0.001; ****P<0.0001. N.S., statistically not significant, with the comparisons labeled. All scale bars = 50 μm.

**Figure 8:**
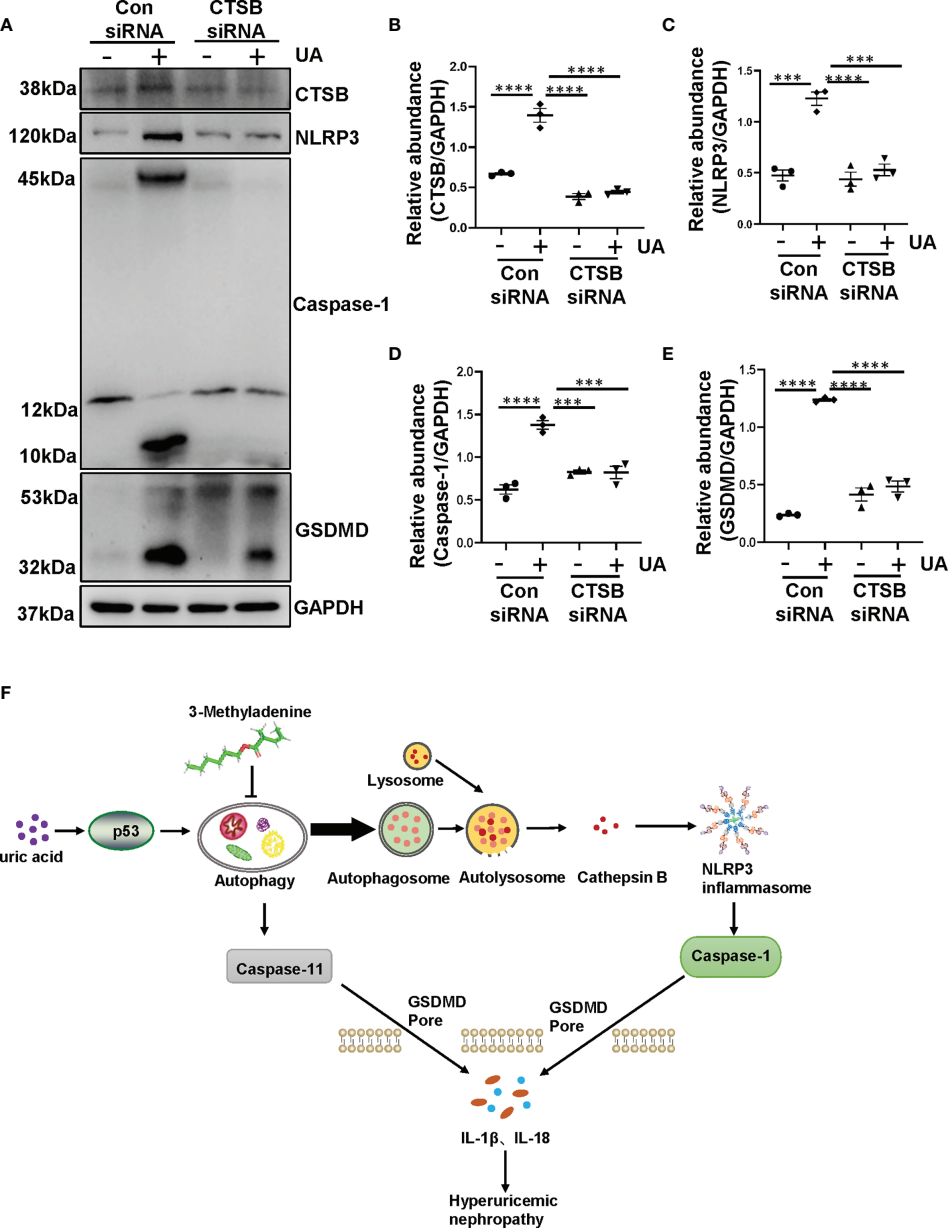
Uric acid-induced NLRP3 inflammasome activation and pyroptosis are regulated by CTSB. **(A)** Western blot was conducted to evaluate the protein level of CTSB, NLRP3, caspase-1, GSDMD and GAPDH in HK-2 cell lysates. **(B–E)** Scatter plots showing the densitometry analysis of CTSB, NLRP3, caspase-1 and GSDMD normalized by GAPDH. **(F)** Mechanisms of uric acid-induced autophagy promotes HN. Chronic exposure to uric acid induces dysregulation of autophagy, which is mediated by p53 pathway. The activation of autophagy further activated NLRP3 inflammasome and pyroptosis. The release of CTSB from autolysosome plays a crucial role in the activation of NLRP3 inflammasome and pyroptosis, which leads to the release of IL-1β and IL-18. Blockade of autophagy inhibits the aforementioned responses so that prevents the occur of hyperuricemic nephropathy. Data are expressed as mean ± SEM. ***P<0.001; ****P<0.0001.

Previous study has indicated that crystal-induced (such as monosodium urate) lysosomal damage plays a crucial role in activating the NLRP3 inflammasome (32). Lysosomes contain several proteolytic enzymes, one of which is the CTSB family. First, we investigated whether CTSB was released into cytoplasm in the kidney of HN rats. Protein level of CTSB significantly increased in HN rats compared to the sham rats, administration with 3-MA reduced its expression (**Figures 7A, B**). Immunohistochemistry staining pointed out that CTSB mainly expressed in the cytoplasm of damaged tubular epithelial cells, and remarkably reduced after 3-MA treatment (**Figures 7C, D**). At the same time, in our *in vitro* study, we found that CTSB expression in HK-2 cells was significantly increased after 36 hours uric acid exposure compared with the sham group, treatment with 3-MA reduced the protein level of CTSB in a dose-dependent manner (**Figures 7E, F**). Immunofluorescence staining confirmed the effect of 3-MA in decreasing the release of CTSB in cytoplasm (**Figures 7G, H**). Consistent with these results, knockdown of Beclin-1 also reduced protein level of CTSB (**Figures 7I, J**). To further examine whether release of CTSB was involved in the activation of NLRP3 inflammasome and pyroptosis, HK-2 cells were transfected with CTSB siRNA to blockage the activities of CTSB. Transfected with CTSB siRNA caused a significant reduction of CTSB protein levels. Knockdown of CTSB had less expression of NLRP3, caspase-1, and GSDMD (**Figures 8A–E**). Taken together, these results demonstrated that CTSB was critical for uric acid-induced activation of NLRP3 inflammasome and pyroptosis, which is associated with autophagy.

In the present study, we found that chronic exposure to uric acid induces dysregulation of autophagy, which is mediated by p53 pathway. The activation of autophagy further activated NLRP3 inflammasome and pyroptosis. The release of CTSB from autolysosome plays a crucial role in the activation of NLRP3 inflammasome and pyroptosis, which leads to the release of IL-1β and IL-18. Blockade of autophagy inhibits the aforementioned responses, thus prevents the development and progression of hyperuricemic nephropathy (**Figure 8F**).

## Discussion

Hyperuricemic nephropathy, a chronic and progressive metabolic disease, is a common clinical complication of hyperuricemia. Our recent study has provided the solid evidence for the renoprotection role of autophagy inhibition in HN (8). However, how does uric acid trigger the activation of autophagy and further mechanism which leads to the HN is not fully investigated. Thus, we further examined the activation of autophagy in uric acid-stimulated HK-2 cells, and investigated the mechanisms by which inhibition of autophagy ameliorates the development of HN induced by feeding a mixture of adenine and potassium oxonate. The present study demonstrated that chronic exposure to uric acid induces activation of autophagy, which is mediated by p53 pathway. Uric acid upregulated the level of autophagy and further triggered NLRP3 inflammasome activation, leading to pyroptotic cell death; and these effects could be inhibited by 3-MA. Additionally, we found that uric acid-induced autophagy was implicated in regulating the release of CTSB, subsequent NLRP3 inflammasome activation, and pyroptotic cell death. Taken together, we have shown that blockade of autophagy prevents HN through inhibiting NLRP3 inflammasome-mediated pyroptosis.

Autophagy is an important physiological process that maintains cellular homeostasis. However, autophagy dysfunction is related to multiple diseases, such as Alzheimer disease and Huntington disease (33, 34). In this study, we found that autophagy was also activated in the uric acid-stimulated HK-2 cells. Images from TEM indicated multiple of autophagy-related vacuoles such as autophagosome and autolysosome were observed in uric acid-stimulated HK-2 cells compared to control cells. Uric acid also triggered a significant upregulation of Beclin-1 and Atg7. These findings are consistent with our previous study, which demonstrated that autophagy was activated in HN rats (8). Despite the emerging evidence showing the upregulation of autophagy during HN, the upstream signaling stimulates the activation of autophagy remains unclear. It is illustrated that p53 activates autophagy (35). p53 is a sequence-specific DNA-binding transcription factor and, among a large number of genes regulated directly by p53, are autophagy-related genes, including Ulk1 and Atg7 (25, 36). p53 induction in this setting contributes to autophagy. We speculated that uric acid also induces autophagy through p53 signaling pathway. In this study, we found that compared to the sham rats, HN rats displayed increased expression of p53 in the kidney. In addition, reduction of p53 expression by its specific siRNA decreased uric acid stimulated expression of Beclin-1 and Atg7. These results demonstrate that uric acid may trigger autophagy by regulating p53 signaling pathway.

Considering that autophagy is activated in HN rats, it is worth to investigate how does autophagy contributes the progression of HN. Emerging evidence have shown that the interplay between autophagy and NLRP3 inflammasome plays a crucial role in metabolic diseases (27, 37). Inflammasome, a complex component of various proteins, is an important part of the innate immune system and plays a key role on detecting the presence of infection, the pathogens and the metabolic signals in cells (38). NLRP3 could be activated by diverse triggers including exogenous pathogen-associated molecular patterns and endogenous damage-associated molecular patterns (39). As a pathogen-associated molecular pattern, uric acid can cause the activation of NLRP3 inflammasome and subsequent release of IL-1β and IL-18 and lead to severe kidney damage (40). In this regard, we found that NLRP3 was significantly increased in HN rats, while administration with 3-MA resulted in the reduction of NLRP3. It suggests that interplay between autophagy and NLRP3 inflammasome plays an important role in the progression of HN. In accordance, uric acid-activated NLRP3 inflammasome is connected with EMT, and tubular interstitial fibrosis (41). These events accelerate HN process. Furthermore, our *in vitro* study also demonstrated that exposure of HK-2 cells to uric acid resulted in increased expression of NLRP3. Interestingly, in HN, monosodium urate, can activate NLRP3 inflammasome in other renal cells. In renal neutrophils and macrophages, NLRP3 inflammasome is activated by monosodium urate crystals which are phagocyted and resulted in the release of proinflammatory cytokines (42). In podocyte, the TXNIP/NLRP3/NF-κB signaling pathway can upregulate the cellular NLRP3 inflammasome expression levels, which is closely related to uric acid-induced podocyte injury (43). In addition, activation of NLRP3 inflammasome is related to interstitial mononuclear cells infiltration and tubular epithelial cells detachment (44). Whether the activation of NLRP3 inflammasomes in these renal cells is regulated by autophagy remains to be further studied.

Recently, the role of autophagy in the activation of NLRP3 inflammasome leading to pyroptosis has been largely investigated. Pyroptosis is a type of inflammatory cell death, GSDMD is believed as the executor of pyroptosis (45). Previous study has demonstrated that caspase-1 and caspase-11 could regulate the process of pyroptosis, which means that the overexpression of caspase-1 and caspase-11 may be the hallmark of pyroptosis (46, 47). Activation of caspase-1 and caspase-11 not only produce IL-1β and IL-18, but also cause cleavage of GSDMD and cell membrane perforation, resulting in the release of various inflammatory factors that aggravate pyroptosis (48). In this study, our results showed that proteins level of caspase-1, caspase-11, and GSDMD markedly increased in HN rats, pretreatment with 3-MA dramatically reduced the elevated expression of caspase-1, caspase-11, and GSDMD in the HN kidney. These results suggested that autophagy plays an important role in contributing the process of pyroptosis in HN. Specifically, pyroptosis is primarily considered to be a unique, proinflammatory cell death in immune cells. However, emerging evidence has shown that pyroptosis is also evoked in other cells, such as alcohol hepatitis–induced hepatocyte pyroptosis, lipopolysaccharide-induced lung endothelial cell pyroptosis, and ischemia/reperfusion–induced primary proximal tubular cells (47, 49, 50). Consistently, our results also demonstrated that caspase-11 and GSDMD were predominantly localized in tubular epithelial cells in injured kidneys associated with HN. Moreover, Uric acid was found to induce a significant upregulation of caspase-1, caspase-11, and GSDMD in HK-2 cells. It is worth noting that the relationship between autophagy and pyroptosis is still controversial. For instance, Pu et al. have indicated that knockdown of Atg7 contributes the activation of inflammasome and pyroptosis in Pseudomonas Sepsis (29). Another study also indicated that inhibition of autophagy can cause NLRP3 inflammasome activation and pyroptosis in dendritic cells or macrophages (51). On the contrary, in benzoapyrene induced HL-7702 human normal liver cells, inhibition of autophagy with 3-MA can ameliorate the pyroptotic cell death (30), which is consistent with the present study. Obviously, the relationship between autophagy and pyroptosis is complicate and further studies are need to address this issue.

Furthermore, the results in the present study may have a significant implication in the cell biology of CTSB. CTSB, a lysosomal cysteine protease, has been demonstrated to participate in autophagy-induced inflammasome activation (52). It has an integral role in autophagy, metabolism, cellular stress signaling, antigen presentation, and lysosome-dependent cell death (53). In our study, protein level of CTSB significantly increased in HN rats compared to the sham rats. Meanwhile, CTSB activation were observed in uric acid-stimulated HK-2 cells. Transfected with the CTSB siRNA results in the reduce of caspase-1 and decrease of pyroptotic cell death in HK-2 cells exposed to uric acid. Furthermore, the increased level of CTSB and subsequent activation of NLRP3 inflammasome and pyroptotic cell death induced by uric acid were reversed by 3-MA. These results demonstrate that CTSB contributes to the uric acid-induced activation of NLRP3 inflammasome, which was autophagy-dependent.

In conclusion, this study demonstrated that chronic exposure to uric acid induces dysregulation of autophagy, which is mediated by p53 pathway. The activation of autophagy further activated NLRP3 inflammasome and pyroptosis. The molecular mechanism of NLRP3 inflammasome activation underlies the autophagy and the release of CTSB from autolysosome caused by uric acid exposure. We also show that blockade of autophagy improves uric acid-induced pyroptosis by inhibiting autophagic-inflammasome pathways so that prevents the occur of hyperuricemic nephropathy. Therefore, this study provides a novel insight into hyperuricemic nephropathy, which may be utilized to improve therapeutic strategies for HN.

## Data Availability Statement

The raw data supporting the conclusions of this article will be made available by the authors, without undue reservation.

## Ethics Statement

The animal study was reviewed and approved by the Institutional Animal Care and Use Committee at Tongji University.

## Author Contributions

NL participated in research design. YH, YS, HC, MT, XZ, JL, YW, and XM conducted experiments. YH, YS, HC, XM, and NL contributed new reagents or analytic tools. YH and YS performed data analysis. YH, YS, and NL wrote or contributed to the writing of the manuscript. All authors contributed to the article and approved the submitted version.

## Funding

This study was supported by the National Nature Science Foundation of China grants (82070791, 81670690, 81470991 and 81200492 to NL), the Outstanding Leaders Training Program of Pudong Health Bureau of Shanghai (PWR12021-02 to NL), the Shanghai Scientific Committee of China (20ZR1445800 and 13PJ1406900 to NL), the Shanghai Health Bureau and Shanghai administration of traditional Chinese Medicine of China (ZHYY-ZXYJHZX-202114 to NL), and the Project of Pudong Health Bureau of Shanghai (PW2021D-04 and PWZxk2017-05 to NL).

## Conflict of Interest

The authors declare that the research was conducted in the absence of any commercial or financial relationships that could be construed as a potential conflict of interest.

## Publisher’s Note

All claims expressed in this article are solely those of the authors and do not necessarily represent those of their affiliated organizations, or those of the publisher, the editors and the reviewers. Any product that may be evaluated in this article, or claim that may be made by its manufacturer, is not guaranteed or endorsed by the publisher.
